# Impact of the Circadian Clock on the Aging Process

**DOI:** 10.3389/fneur.2015.00043

**Published:** 2015-03-06

**Authors:** Sara S. Fonseca Costa, Jürgen A. Ripperger

**Affiliations:** ^1^Department of Biology/Biochemistry, University of Fribourg, Fribourg, Switzerland

**Keywords:** aging, circadian clock, genetic models, regulatory networks, metabolism

## Abstract

The increase of life expectancy and the decline of biological functions with advancing age are impending obstacles for our society. In general, age-related changes can be separated into two processes. Primary aging is based on programs governing gradual changes which are generally not harmful. On the other hand, secondary aging or senescence is more aleatory in nature and it is at this stage that the progressive impairment of metabolic, physiological, and neurological functions increases the risk of death. Exploiting genetic animal models, we obtain more and more information on the underlying regulatory networks. The aim of this review is to identify potential links between the output of the circadian oscillator and secondary aging. The reasons to suspect such links rely on the fact that the mouse models without functional circadian clocks sometimes exhibit reduced life expectancy. This may be due to their inability to properly control and synchronize energy expenditure, affecting, for example, the integrity of neurons in the brain. Hence, it is tempting to speculate that re-synchronization of metabolic and physiological functions by the circadian clock may slow down the aging process.

## Introduction

Aging can be regarded as a progressive functional decline or deterioration of physiological functions (1). This intrinsic, inevitable, and currently irreversible process increases the vulnerability of an organism and consequently enhances the loss of viability (2, 3). In principle, aging can be described by two independent but connected processes. Primary aging describes the gradual process of body deterioration that takes place throughout life. It was demonstrated that primary aging is partly based on genetic programs, and consequently seems pre-programed. The second process is referred to as secondary aging or senescence, which likely results from external factors such as disease, lack of physical activity, unhealthy activities (e.g., excessive smoking and drinking), poor nutrition, and exposure to hazardous materials (4). How secondary aging impacts primary aging is actually unknown. Hence, secondary aging is considered more haphazard and therefore, a difficult process to characterize (2).

**Figure 1:**
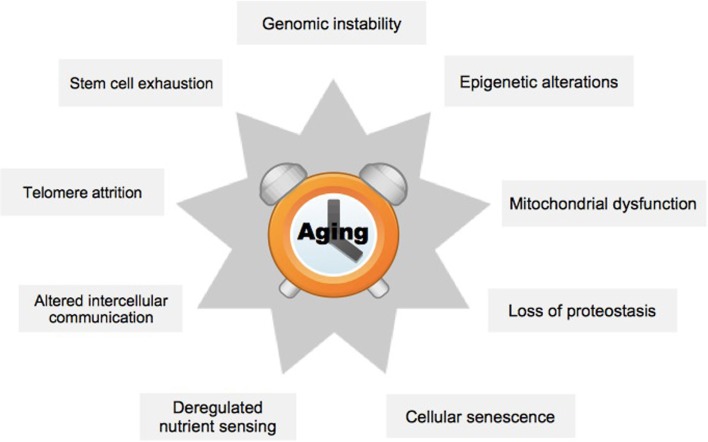
**The nine hallmark processes of aging**. The scheme summarizes hallmark processes typically affected by aging, see text. Adapted from López-Otín et al. (1).

In order to better understand the effects of aging on mammals, nine hallmark processes have previously been defined (Figure 1) (1). These encompass metabolic or regulatory changes within a cell (cellular level), or problems in the interaction of cells (organ level). The hallmark process of genomic instability is characterized by increased mutation rate, which may lead to inappropriate expression of target genes (5). A specific case occurs at the ends of the chromosomes, the telomeres, which diminish in length with each cell generation, ultimately causing chromosome aberrations (6). Epigenetic alterations group processes that affect target gene expression without changing the underlying genetic code, for example, by altered DNA methylation (7). Loss of proteostasis refers to the increasing inability of cells to remove misfolded proteins and other debris leading to the accumulation of toxic products (8). Deregulated nutrient-sensing affects signaling cascades within cells, which normally regulate the balance of metabolic activity and rest (9). Mitochondrial dysfunction may be due to damage from oxygen radicals produced during the process of oxidative phosphorylation (10). Cellular senescence restricts the replicative life of a cell and has previously been validated as a primary mediator of aging (11). Impairment of stem cells is supposed to explain aging of tissues because stem cells are essential for maintaining tissue homeostasis, and loss of these cells leads to a breakdown of functions in organs, for example, in the forebrain (12). Finally, inflammation and consequently perturbed communication between the individual cells within an organ may cause substantial damage to its overall functioning (13).

In this review, we would like to relate some aspects of the hallmark processes of aging to the circadian clock (14, 15). The circadian clock in mammals is based on interconnected transcriptional feedback loops, which are fine-tuned by post-translational regulation to generate rhythms with a periodicity of about a day (16). The circadian clocks are ubiquitous throughout the body and help us to regulate rhythmic processes in metabolism and physiology. Many age-related phenotypes observed in mice without functional circadian clocks suggest that the circadian clock may be able to counteract the aging process.

## Circadian Rhythms and the Hallmarks of Aging

### Genomic instability

Genomic instability results in the loss of genetic information and in the worst case scenario activates genes favorable for tumor formation (17). To prevent this phenomenon, some genes act as tumor suppressor proteins to maintain genomic stability, for example, the NAD^+^-dependent deacetylase SIRT6 (18). This enzymatic activity, when overexpressed, extended the lifespan of mice (19), likely by direct interaction with the chromatin remodeler Snf2h at DNA repair sites (20). Recently, it was shown that there is a direct interaction of SIRT6 with the circadian regulators BMAL1 and CLOCK adding a circadian component to its activity (21). Furthermore, the rate-limiting enzyme for NAD^+^ synthesis, NAMPT, was shown to be linked to the circadian oscillator by a similar mechanism (22, 23), and to decline in SCN neurons with age (24). Concomitantly, the cofactor required for the functioning of SIRT6 decreases in the brain. Hence, it is tempting to speculate that the activity of SIRT6 in the nucleus is affected by the circadian oscillator with age and as such the circadian oscillator may adversely impact genomic stability.

### Telomerase activity

Telomeres are characteristic repetitive sequences at the end of chromosomes, whose length has to be reconstituted after each cell division by a specific enzyme, the telomerase (TERT) (6). Their main function is to prevent the loss or rearrangement of chromosomes. Surprisingly, in humans, telomerase activity declines with age limiting the replicative life and affecting function of cells. For example, if telomere shortening occurs in hematopoietic stem cells (25), their function and engraftment ability are significantly compromised. Interestingly, the enzyme TERT and its activity were found to be under circadian control in mice. Circadian expression of TERT mRNA is hardwired to the circadian oscillator via direct regulation by the BMAL1 and CLOCK heterodimer (26). Mice deficient for CLOCK do not display rhythmic telomerase activity and their chromosomes have shorter telomeres. On the other hand, it was shown that reconstitution of TERT into senescent fibroblasts could reconstitute their circadian system (27). Taken together, the interplay between TERT and the circadian oscillator may contribute to aging.

### Epigenetic alterations

Epigenetic changes are inherited from one cell generation to the next without changing the underlying genetic code (7). This may be achieved by transferring specifically modified histones or DNA methylation patterns to the next generation. Both kinds of epigenetic changes may activate or repress neighboring genes. As an example for the impact of the circadian oscillator on the epigenetic histone modification machinery, the NAD^+^-dependent histone deacetylase SIRT1 is implicated in circadian gene regulation by enhancing *Bmal1* and *Clock* transcription (28, 29). This enzymatic activity declines in the brain with age (24), which may directly impact the functioning of the circadian oscillator and feed back on *Sirt1* expression. Many other histone modifying enzymes have been linked to the circadian oscillator as well (30). As an example for the effect of changing methylation patterns, oxidation of 5-methyl-cytosine to 5-carboxyl-cytosine changed the binding activity for the Wilms Tumor protein with age (31). The binding site for BMAL1 and CLOCK, 5′-CACGTG-3′, may be sensitive to a similar phenomenon with age, because it contains the cytosine methylation motif 5′-CG-3′ in its center. A potential concomitant reduction in DNA binding would mimic a loss of BMAL1 and CLOCK function and mice deficient in *Bmal1* or *Clock* exhibit a significantly reduced life expectancy (32, 33), implicating a function of these factors in the aging process. Here, again we find a potential impact of the circadian oscillator on very basic regulatory processes in the cell.

### Loss of proteostasis

With age damaged and toxic products accumulate in the cells (8). These may be degradation products of proteins, misfolded proteins due to impaired function of chaperones, or xenobiotic substances. Under normal circumstances, cells can handle this debris and even have the potential to repair some of the damage. Unfortunately, during the aging process, the function of many of these repair pathways declines, which causes accumulation of unwanted and mostly useless materials. Although some repair mechanisms in the cell, for example, the removal of the methyl group from *O*^6^-methyl-guanosine (34), are under control of the circadian clock, a direct influence on the age-related degradation of proteostasis is not yet illuminated. However, part of the detoxification program of the liver is under control of the circadian PAR-bZip transcription factors (35). Mice deficient in these transcription factors are sensitive to xenobiotic substances and have about half the life expectancy of normal mice. A mechanistic link between the PAR-bZip transcription factors and aging remains to be established, though. Nevertheless, it is thought that there is at least an indirect effect of the circadian oscillator on proteostasis in the brain, because the circadian oscillator regulates the balance of metabolic processes in the cell, which may become disturbed with increasing age (36).

### Nutrient sensing

Caloric restriction is an established mean to prolong life span of a variety of organisms by influencing the metabolic activity of cells (9). The first longevity mutants isolated from *C. elegans* identified the insulin and IGF-1 signaling pathway important for nutrient sensing and longevity (37). Mice with disrupted circadian clock are often prone to metabolic syndrome due to deregulated metabolic pathways and concomitantly display insulin resistance (38), which impairs nutrient sensing. Hence, the disruption of the circadian clock, or a simple misalignment of the circadian clock with the environment, may reduce life expectancy. This was verified in a simple experiment that correlated the life expectancy of mice to the precision of their circadian clock in maintaining 24 h periodicity under constant conditions (39). Interestingly, insulin affects transcription of the *Clock* gene and hence feeds back on the circadian oscillator (40). Another pathway that is affected by the circadian clock and senses the availability of nutrients is the target of rapamycin (TOR) pathway (41). TOR signaling is high in *Bmal1*-deficient mice, which is in agreement with their reduced life expectancy. Administration of the TOR-signaling inhibitor rapamycin can increase the life expectancy of *Bmal1*-deficient mice by up to 50%. Consequently, it is feasible that at least some part of the reduced life expectancy of *Bmal1*-deficient mice is due to a malfunction of nutrient-sensing TOR-signaling.

### Mitochondrial dysfunction

Mitochondria are power-generating organelles and are the place of some of the most aggressive oxidative reactions within the cell (10). Due to this oxidative microenvironment, somatic mutations of the mitochondrial genome are common, which ultimately impairs its function. The circadian clock may be linked to the aging of the mitochondria via the NAD^+^-metabolism (42). This specific metabolic pathway is under the direct control of the circadian clock via NAMPT (22, 23) and NAD^+^ affects the activity of the NAD^+^-dependent deacetylase SIRT3 (42). This enzymatic activity rhythmically regulates, by deacetylation, the activities of many metabolic enzymes located in the mitochondria. In general, acetylation diminished, while deacetylation increased the activities of mitochondrial enzymes involved in oxidative phosphorylation. In *Bmal1*-deficient mice, due to the lack of NAD^+^, SIRT3 activity was diminished and consequently the energy metabolism reduced. Most importantly, this effect could be rescued by the administration of NMN, a NAD^+^ precursor, to the *Bmal1*-deficient mice. Hence, the connection between the NAD^+^ metabolism and the activity of mitochondrial enzymes is well established. This connection suggests a direct effect of the circadian oscillator on the aging process of mitochondria.

### Cellular senescence

Cellular senescence refers to the observation that cells kept in culture only have limited replication performance. This phenomenon was already described more than 50 years ago and called the Hayflick limit (43). Previously, it was shown that cellular senescence was a major contributor to the aging of an organism and may be partly due to the shortening of telomeres. A molecular link to the circadian oscillator, for example, is provided by the NOPS-family of transcriptional regulators. NONO, one of the members of this family, was shown to interact with the circadian repressor protein Period1 and as such to affect circadian rhythms (44). Cells derived from *Nono*-deficient mice show an advanced senescence phenotype (45). This may be due to direct interference with the cell cycle via circadian regulation of the p16-Ink4A gene, which is implicated in senescence. However, it is not known yet, whether *Nono*-deficient mice have a reduced life expectancy. Surprisingly, mice deficient for *Bmal1* have an increased number of senescent cells *in vivo* but not *in vitro* compared to their litter mate controls (46). Further analysis revealed that *Bmal1*-deficient cells are hypersensitive to damaging stress, for example, due to the generation of radical oxygen species. Hence, the phenotype observed in *Bmal1*-deficient mice may be due to problems in damage control rather than cellular senescence *per se*. Nevertheless, it is conceivable that the circadian oscillator also affects cellular senescence either directly or indirectly.

### Stem cell exhaustion

Stem cells are important to keep homeostasis of tissues by replenishing cells lost due to damage (12). Only a small number of stem cells, however, have to differentiate into new cell types, while the remaining have to self-renew their population. With age, a reduction of stem cells is observed, which may affect the maintenance of tissue function. Previously, it was demonstrated that the circadian clock affects the equilibrium between self-renewal and differentiation of epidermal stem cells (47). Because these cells are not all in the same circadian phase, only a subset of stem cells in this particular niche react to signals to become activated. Interestingly, in *Bmal1*-deficient mice, there was accumulation of dormant stem cells, while in *Period1*/*Period2*-deficient mice there was depletion of this kind of cells. The phenotype of arrhythmic stem cells caused premature aging of the epidermis in *Bmal1*-deficient mice. These results indicate that the circadian clock fine-tunes the temporal behavior of epidermal stem cells. Consequently, perturbation of the delicate equilibrium of stem cells with age affect homeostasis and tissue function (48).

### Intercellular communication

Intercellular communication allows for synchronization of the entire population of cells within a tissue (13). The impact of aging on this process involves local inflammation and the concomitant communication of tissue and immune cells by cytokines and other mediators. Previously, it has been demonstrated by parabiosis experiments that factors in the blood of old mice could impair cognitive functions of young mice. This happened likely by increasing local neuroinflammation due to the increased release of chemokines (49). Interestingly, blood taken from old humans impaired the function of the circadian oscillator in fibroblasts (50). However, the performance of the circadian oscillators in fibroblasts derived from either young or old individuals was indistinguishable, prompting to a yet to be identified signaling cascade responsible for the deteriorating effect. Similarly, the function of the suprachiasmatic nucleus, the master circadian clock in the brain of mammals, became obstructed with age not at the molecular, but at the neuronal output level affecting the communication of neurons in the suprachiasmatic nucleus (51). This observation was recently verified in very old mice, in which the phase-synchronization between the individual SCN neurons gets abolished, directly affecting the rhythmicity of the mice (52). Taken together, it is well imaginable that some circadian control of intercellular communication within the brain is changed with increasing age with adverse effects on the overall functioning of the neuronal networks.

## Conclusion

Here, we provided only a handful of potential molecular links between the circadian oscillator and aging. The circadian clock synchronizes metabolism and physiology of an organism to enhance fitness and to optimize energy expenditure. Consequently, it optimizes the functioning of an organism by helping to avoid damage to its cells and the accumulation of toxic products. Unfortunately, the circadian clock is not resilient to the aging process and its synchronization abilities steadily decline. On the other hand, a function for the aging processes is not known. Probably, it is just not necessary to maintain an organism in perfect shape after arresting its reproductive potential. Suicide programs similar to those eliminating superfluous cells in the body such as apoptosis have not yet been described to affect life expectancy. However, both tendencies together (decline of the circadian clock and increasing age-related changes) yield an accumulation of damage which finally increases the risk of death. Hence, if it is possible to resynchronize the circadian clock in an old organism and to reconstitute at least part of the damage-controlling programs it may be possible to circumvent age-related problems, for example in the brain.

## Conflict of Interest Statement

The authors declare that the research was conducted in the absence of any commercial or financial relationships that could be construed as a potential conflict of interest.
